# Association of adverse childhood experiences and health risk behaviors among young adults visiting a regional primary healthcare center, Federation of Bosnia and Herzegovina

**DOI:** 10.1371/journal.pone.0194439

**Published:** 2018-03-29

**Authors:** Sanjin Musa, Corrine Peek-Asa, Nina Jovanović, Edin Selimović

**Affiliations:** 1 Department of Epidemiology, Institute of Public Health of the Federation of Bosnia and Herzegovina, Sarajevo, Bosnia and Herzegovina; 2 Department of Occupational and Environmental Health, University of Iowa, Iowa City, Iowa, United States of America; 3 Department of Ophthalmology, County Hospital Zenica, Zenica, Bosnia and Herzegovina; 4 Department of Dentistry, Healthcare Center of Zenica, Zenica, Bosnia and Herzegovina; Harvard Medical School, UNITED STATES

## Abstract

**Background:**

Many studies have linked adverse childhood experiences (ACEs) to long-term health outcomes, as well as health risk behaviors. In the post-war period in Bosnia and Herzegovina, many young people grew up in an environment of deteriorated living standards due to high unemployment and economic insecurity. The objectives of the study were to: 1) describe the health risk behaviors of young adults accessing primary healthcare; and 2) examine associations of these risk factors with adverse childhood experiences in this context.

**Methods:**

This was a cross-sectional survey, conducted from April to October 2014. Participants were recruited from the Primary Healthcare Center Zenica. Patients between the ages of 18 and 24 were eligible for inclusion. The informed consent and self-administered questionnaire were offered to patients during clinic intake. The questionnaire contained questions on sexual and reproductive health, use of alcohol and drugs, dating violence, and adverse childhood experiences.

**Results:**

During the study period 520 questionnaires were distributed, and 400 complete surveys were returned, for a response rate of 76.9%. Among the 400 respondents, 166 were males (41.5%) and 234 were females (58.5%). Our study showed that 48.7% of respondents had experienced some form of childhood adversity. Emotional neglect was the most common type of adverse childhood experience (25.6%) and was significantly more prevalent among females. Our study indicated that more than 15% of respondents had witnessed domestic violence. Overall, ACEs were associated with increased odds of early sex initiation, alcohol use, drug use, and dating violence, although some of these associations did not reach statistical significance. Emotional neglect was the exception, and reporting emotional neglect was associated with a significantly elevated odds ratio for all four of the health risk behaviors. Emotional abuse was associated with an increased odds of drug abuse (OR = 2.78; 95% CI = 1.31–5.90) and dating violence (OR = 2.31; 95% CI = 1.10–4.89). Sexual abuse was marginally associated with increased early sex initiation (OR = 3.2; 95% CI 0.93–10.8). Parental divorce was significantly associated with alcohol abuse.

**Conclusion:**

The results of this study demonstrated associations between adverse experiences in childhood and the probability of engaging in health risk behavior which has implications for health outcomes in the long-term.

## Introduction

Every year throughout the world, millions of children are victims of adverse experiences that create toxic stress, including non-fatal abuse and neglect [1]. One of the first studies linking Adverse Childhood Experiences (ACEs) to premature death was conducted by a large healthcare provider in San Diego, California [2]. This study revealed a relationship between children's emotional experiences and adult's emotional and physical health. Since then many studies have linked ACEs to long-term health outcomes including ischemic heart disease, cancer, chronic lung disease, suicide, and skeletal fractures as well as health risk behaviors such as substance use and smoking [3–11]. ACEs have been associated with indicators such as poor school performance and unemployment [12]. Studies have also linked ACEs with a range of adolescent risk behaviors such as violence perpetration and sexual aggression in adolescent dating relationships, sexual risk behaviour, and adolescent mental health [13–16].

The basic mechanism tying ACEs to poor health outcomes lies in the permanent shifts in neurological development induced by toxic stress. Preclinical and clinical studies confirm that severe stress early in life induces persistent maladaptations in multiple neurotransmitter systems involved in the regulation of stress, specifically characterized by enhanced behavioral, neuroendocrine and autonomic stress response [17]. Consideration of the positive neuroregulatory effects of health risk behaviors such as alcohol or drug abuse may provide bio-behavioral explanations for the link between adverse childhood experiences and health risk behaviors and diseases in adults [18]. Adolescence/youth is often characterized as a stage of biological and social transitions with a range of risky behaviors, including sexual risk behaviors, alcohol and substance use as well as peer violence [19–22]. However, interventions at the individual, family, and community level that support resilience and recovery from adverse events can positively impact health trajectories [23,24,25]. Identifying risk factors and populations at risk are a foundation of focusing prevention and intervention efforts.

Epidemiologic studies demonstrate that risk factors for ACEs and their related health and social outcomes are not randomly distributed in the population [3–11]. In 2009, the World Health Organization and U.S. Centers for Disease Control and Prevention (CDC) launched an effort to build a framework of public health surveillance with the goal of determining the global burden of adverse events from childhood [26]. This framework acknowledges that sources of adverse events are many, and that combinations of individual and community-level factors contribute to toxic stress and its outcomes.

In the post-war period in Bosnia and Herzegovina (1995–2016), many young people grew up in an environment of deteriorated living standards due to high unemployment and economic insecurity. The country has experienced major political, social and economic changes in recent decades. Despite the significant reconstruction of society and economic recovery, young people remain a vulnerable group. Few studies in the Balkan region have examined the incidence of ACEs, and these existing studies identify relatively high rates [27,28]. Adolescent cohorts in this region are of interest due to the impact of the war on their families and communities. Studies in the region have not tied health risk behaviors with individual ACEs, and we hypothesize that this relationship could be stronger within a cohort of youth who were war-impacted as children. Therefore, the aims of the study were to 1) describe health risk behaviors of young adults accessing primary healthcare in Bosnia and Herzegovina; and 2) examine the associations of risk behaviors with adverse childhood experiences.

## Materials and methods

The study protocol was reviewed and approved by the Ethics Committee of the Healthcare Center of Zenica and the Ethics Committee of the Medical Faculty University of Sarajevo. This was a cross-sectional survey conducted from April to October 2014, based on a convenience sample. Our power calculations indicated the need to recruit 520 respondents. Based on previous research, we estimated the prevalence of early sexual activity as 3.9% among those reporting no adverse experiences and 11.4% among those reporting at least two adverse experiences [27,28]. Using an alpha of 0.05, an expected difference of 7.5%, and power of 0.8, we calculated a final sample size of 392. The final recruitment sample size accounts for a non-response rate of 30%.

Participants were recruited from the Primary Healthcare Center Zenica, which is one of the largest primary healthcare institutions in the Federation of Bosnia and Herzegovina with 150,000 registered patients. The following departments were included in the survey: Family medicine, Occupational medicine, Department of women’s health, Dental service and Center for mental health. The health care system in Bosnia and Herzegovina is largely supported through government resources, which fund the primary health care centers. These centers only provide outpatient care which includes a wide variety of specialist services.

Patients between the ages of 18 and 24 were eligible for inclusion. Recruitment included patients referred from community-based family physicians’ practices or presented at the Primary Healthcare Center Zenica for a standardized psychosocial or medical evaluation and occurred during business hours (7–19) on weekdays (Monday through Friday) over a period of 6 months. Participation in the survey was voluntary, and all potential participants were informed of the aims of the survey. The informed consent and self-administered questionnaire were offered to patients during clinic intake. Research assistants approached eligible young patients in the waiting room.

The questionnaire was created based on the Adverse Childhood Experiences (ACE) questionnaires, KAPB (knowledge, attitude, practice and beliefs) questionnaire developed in the study in Croatia (Štulhofer et al. 2010), *Behavioral Risk Factor Surveillance System Questionnaire* and Bonomi's surveys [29,30,31]. The survey instrument contained questions on sexual and reproductive health, use of alcohol and drugs, dating violence, and adverse childhood experiences. The ACE questionnaire was translated from English to the local language. The first draft of the questionnaire was tested in a pilot study. Four questions were removed from the final form of the questionnaire. Survey administrators were nurses instructed and supervised by a research field coordinator. Each questionnaire was administered in a confidential environment and took approximately 25 minutes in duration.

### Measures

Dependent variables included risky sexual behavior, dating violence, and use of alcohol/drugs. Risky sexual behavior was defined as early initiation of sexual activity (16 years of age or younger): *Have you ever had sexual intercourse*? *How old were you when you first had sexual intercourse*? Dating violence included physical, sexual, and psychological violence by a dating partner, and questions were adapted from existing research [31]: *Have you ever been in a relationship with someone who hit you*, *slapped you or physically hurt or threatened to hit or slap you*, *destroyed something that belongs to you*? *Have you ever been in a relationship with someone who forced you to have sexual intercourse by arguing with you or threatening you with physical force*? *Have you ever been in a relationship with someone who tried to control your behaviour by always checking up on you*, *called you names*, *said things to hurt you*, *shouted at you*, *made unwanted calls*, *text messages or emails*? Dating violence was dichotomized to include those with any positive versus all negative responses. Use of alcohol and drugs included any self-reported experience with alcohol/substance abuse: *During the past 30 days*, *on how many days did you have at least one drink of alcohol*? *During the past 30 days*, *on how many days did you have 5 or more drinks of alcohol in a row*, *that is*, *within a couple of hours*? *During your life*, *how many times did you use marijuana*? *During the last month*, *how many times did you use marijuana*? *Have you ever used drugs intravenously*? *Have you ever used other types of drugs*: *cocaine*, *sniffed glue*, *methamphetamines (speed)*, *ecstasy*, *pills (without a doctor’s prescription)*?

Independent variables measured adverse experiences [27,28,29] of participants during the first 18 years of life, including 8 questions assessed on a 5-point Likert scale (0 = *never* to 4 = *very frequent)* and 6 questions assessed with positive or negative responses. A number of factors were included in this list of ACEs and were defined as follows. For domestic violence, participants were asked: *Was your mother or stepmother often or very often pushed*, *grabbed*, *slapped*, *or had something thrown at her*? *Was your mother or stepmother ever repeatedly hit at least a few minutes or threatened with a gun or knife*? A response of “sometimes,” “often” or “very often”indicated that a respondent was exposed to domestic violence. The following question was posed for household substance abuse: *Did you ever live with someone who was a problem drinker or alcoholic or who used street drugs*? For parental separation or divorce respondents were asked: *Were your parents ever separated or divorced*? For exposure to incarceration, the question asked was: *Did a household member go to prison*? Regarding mental illness, respondents were asked: *Was a household member depressed or mentally ill*, *or did a household member attempt or commit suicide*?

Questions related to abuse and neglect were also included in the assessment of ACEs. For emotional abuse the following questions were asked: *How often did a parent or other adult in your household swear at you*, *insult you*, *put you down or humiliate you*? *How often did a parent or other adult in your household act in a way that made you afraid that you might be physically hurt*? Responses of “often” or “very often” to either item defined emotional abuse during childhood. Questions concerning physical abuse included. *How often did a parent or other adult in the household push*, *grab*, *slap*, *or throw something at you*? Respondents were defined as experiencing physical abuse if they answered “often” or “very often”. For sexual abuse, the following questions were asked: *Did an adult or person at least 5 years older than you ever touch or fondle you or have you touch their body in a sexual way*? *Did an adult or person at least 5 years older than you attempt or actually have oral*, *anal*, *or vaginal intercourse with you*? A “yes” reply to any of these two questions defined a participant as having experienced sexual abuse during childhood. Questions on emotional neglect were: *How often did you feel that no one in your family loved you or thought you were important or special*? *How often did you feel that your family didn’t look out for each other*, *feel close to each other*, *or support each other*? Responses of “often” or “very often” to either item defined emotional neglect during childhood. For physical neglect, included questions were: *How often did you feel that you didn’t have enough to eat*, *had to wear dirty clothes*, *or had no one to protect you*? Responses of “often” or “very often” defined physical neglect during childhood.

### Analytic strategy

Data were entered into Microsoft Access where they were coded and cleaned, and then exported to SPSS ver. 17.0 for analysis (SPSS, Inc., 2009, Chicago, IL, USA). Univariate examination of frequencies, percentages, means, and standard deviations were examined for each study variable. Significant differences between the sub-samples were calculated using the Student's t-test and Mann-Whitney U test for interval or continuous variables, and Pearson chi-square test (with Yates' correction when necessary) for categorical variables.

The association between adverse childhood experiences and risk behavior engagement was estimated using logistic regression. Logistical regression was done between the components of ACE and indicators of risky behavior related to sexual and reproductive health to estimate odds ratios and 95% confidence intervals. Multiple logistical regression analysis was employed to statistically control for potential confounding variables when analyzing the relationship between exposure to ACE and risky sexual behaviors. Confounding variables were identified based on previous literature and through an existing association with the exposure and outcome variables. Adjusted odds ratios and 95% confidence intervals (CIs) were estimated to examine the relationships between the number of types of exposure to adverse childhood experiences and selected health risk-taking behaviors. Odds ratios were adjusted for sex, age, socioeconomic status, residence status, relationship status, and occupational status.

## Results

### Response rate and sociodemographic characteristics

During the study period 520 questionnaires were distributed, and 400 complete surveys were returned, for a response rate of 76.9%. Among the 400 respondents, 166 were males (41.5%) and 234 were females (58.5%). The average age of respondents was 20.2 years for males and 20.6 years for females (Table 1).

**Table 1:** Sociodemographic characteristics of respondents.

Demographic and socioeconomic characteristic of respondents	Male N(%)	Female N(%)	Total N(%)	Significance (χ^2^, df, p-value*)
**Sex**	166 (41.5%)	234 (58.5%)	400(100.0%)	
**Age, Mean (SD)**	20.2 (2.3)	20.6 (2.0)	20.4 (2.2)	t-test -1.749; p = 0.081
**What was the size of the city where you have lived for most of your life?**				9.315; 4; 0.054
Less than 10,000 inhabitants	18 (11.0%)	20 (8.5%)	38 (9.5%)	
10,001–50,000 inhabitants	25 (15.2%)	19 (8.1%)	44 (11.1%)	
50,001–100,000 inhabitants	41 (25.0%)	86 (36.8%)	127 (31.9%)	
100,001–500,000 inhabitants	71 (43.3%)	96 (41.0%)	167 (42.0%)	
More than 500,000 inhabitants	9 (5.5%)	13 (5.6%)	22 (5.5%)	
**Assess your financial situation (i.e. the financial situation for most of your family members):**				1.542; 4; 0.819
Much worse than others	18 (10.8%)	31 (13.3%)	49 (12.3%)	
Slightly worse than most others	22 (13.3%)	33 (14.2%)	55 (13.8%)	
Neither better nor worse than most others	61 (36.7%)	88 (37.8%)	149 (37.3%)	
Slightly better than most others	44 (26.5%)	59 (25.3%)	103 (25.8%)	
Much better than most others	21 (12.7%)	22 (9.4%)	43 (10.8%)	
**Are you currently:**				3.637; 2; 0.162
Married	24 (14.5%)	38 (16.3%)	62 (15.5%)	
ln a relationship	43 (25.9%)	78 (33.5%)	121 (30.3%)	
Not in a relationship	99 (59.6%)	117 (50.2%)	216 (54.1%)	
Are you currently:				16.143; 3; 0.001*
Secondary school student	71 (42.8%)	68 (29.1%)	139 (34.8%)	
College student	32 (19.3%)	66 (28.2%)	98 (24.5%)	
Employed	35 (21.1%)	34 (14.5%)	69 (17.3%)	
Unemployed	28 (16.9%)	66 (28.2%)	94 (23.5%)	

*p < .05

Nearly half (47.5%) of respondents reported living for the majority of their lives in a city with 100,000 inhabitants or more. Just over a third (37.3%) reported having a financial situation no different from others, with 36.6 reporting being better off and 26.1% indicating being worse off. More than a half of the sample were secondary school and college students (59.3%) and reported not being in a relationship (54.1%). More female than male respondents reported being married or in a relationship and not being employed.

### Adverse childhood experiences

Emotional abuse (24.5%) and emotional neglect (25.6%) were the most common adverse childhood experiences, and both were reported more frequently by females than males (Table 2). Physical abuse and neglect were reported by approximately the same number of respondents (10.3% and 11.0% respectively), and these were also reported significantly more often by females than males. Sexual abuse was the least frequently reported type of child abuse and neglect, reported by 7.1%, and the only type of abuse and neglect that was not reported by a significantly higher proportion of females.

**Table 2:** Adverse childhood experiences.

Adverse Childhood Experience	Male N(%)	Female N(%)	Total N(%)	Significance (χ^2^, df, p-value*)
**Exposure to emotional abuse**	29 (17.5%)	69 (29.5%)	98 (24.5%)	6.964; 1; 0.008*
**Exposure to emotional neglect**	29 (17.6%)	73 (31.3%)	102 (25.6%)	8.880; 1; 0.003*
**Exposure to physical abuse**	7 (4.2%)	34 (14.5%)	41 (10.3%)	10.134; 1; 0.001*
**Exposure to physical neglect**	9 (5.5%)	35 (15.0%)	44 (11.0%)	7.964; 1; 0.005*
**Explosure to sexual abuse**	8 (4.8%)	20 (8.6%)	28 (7.1%)	1.557; 1; 0.212
**Exposure to domestic violence**	21 (12.7%)	44 (18.9%)	65 (16.3%)	2.248; 1; 0.134
**Substance abuse by family member**	15 (9.1%)	45 (19.4%)	60 (15.1%)	7.199; 1; 0.007*
**Mental illness of family member**	11 (6.7%)	19 (8.1%)	30 (7.5%)	0.122;1; 0.727
**Family member in prison**	7 (4.2%)	10 (4.3%)	17 (4.3%)	0.000;1; 1.000
**Parents divorced or separated**	28 (17.0%)	68 (29.1%)	96 (24.1%)	7.094;1; 0.008*
**Number of ACEs experienced**				17.055;4; 0.002*
**0**	101 (61.2%)	101 (44.1%)	202 (51.3%)	
**1**	20 (12.1%)	31 (13.5%)	51 (12.9%)	
**2**	16 (9.7%)	33 (14.4%)	49 (12.4%)	
**3**	15 (9.1%)	18 (17.9%)	33 (8.4%)	
**4+**	13 (7.9%)	46 (20.1%)	59 (15.0%)	

*p < .05

Parental divorce or separation was the most common type of familial adverse experience reported, with a prevalence of 24.1%. Exposure to domestic violence (16.3%) and substance abuse by a family member (15.1%) were the next two most prevalent familial adverse experiences. Parental divorce and substance use by a family member were significantly higher among females. Mental illness (7.5%) or incarceration (4.3%) of a family member were the least prevalent and did not differ significantly by gender.

Overall, approximately half of respondents reported no ACEs, while 15.0% of respondents reported four or more and 8.4% reported three. Females reported a much higher cumulative number of ACEs, with 20.1% reporting four or more ACEs and 17.9% reporting three. In contrast, 61.2% of males compared with 44.1% of females reported no ACEs.

### Sexual activity and dating violence

Slightly more than half (51.0%) of respondents reported that they were sexually active, with no difference between males and females (Table 3). The average age of sexual debut was 18.7 years, with no variation by gender (male = 18.8 and female = 18.7). Sexual debut before or at the age of 16 years was reported by 9.5% of participants, and this was considered to be risky sexual behavior.

**Table 3:** Frequency of health risk behaviors by gender: Early sex initiation and dating violence.

Sexual experiences	Male N(%)	Female N(%)	Total N(%)	Significance(χ^2^, df, p-value*)
**Having sexual intercourse**				0.055; 1; 0.814
**Yes**	83 (50.0%)	121 (51.7%)	204 (51.0%)	
**No**	83 (50.0%)	113 (48.3%)	196 (49.0%)	
**Sex initiation 16 years of age or younger**				2.183; 1; 0.140
**Yes**	11 (13.3%)	27 (22.5%)	38 (18.7%)	
**No**	72 (86.7%)	93 (77.5%)	165 (81.3%)	
**Physical violence**				23.173; 1; 0.000*
**Yes**	6 (3.7%)	49 (21.3%)	55 (14.0%)	
**No**	157 (96.3%)	181 (78.7%)	338 (86.0%)	
**Sexual violence**				27.013; 1; 0.000*
**Yes**	2 (1.2%)	43 (18.7%)	45 (11.5%)	
**No**	161 (98.8%)	187 (81.3%)	348 (88.5%)	
**Psychological violence**				15.296; 1; 0.000*
**Yes**	36 (22.0%)	95 (41.3%)	131 (33.2%)	
**No**	128 (78.0%)	135 (58.7%)	263 (66.8%)	

*p < .05

Psychological violence was the most prevalent form of dating violence reported with a prevalence of 33.2% for the total sample. Physical violence was reported by 14.0% and sexual violence by 11.5% of respondents. For all three types of dating violence, females reported a significantly higher prevalence than males. Physical violence was nearly five times more prevalent among females than males, and sexual violence was nearly fifteen times more prevalent. Psychological violence was just under twice as frequent among females as compared to males.

### Alcohol and drug use

In the 30 days prior to the survey, 31.8% of respondents reported alcohol use (Table 4). Consuming twenty or more drinks was reported by 2.0% of respondents, and 15.9% reported consuming 5 or more drinks at one time. There were no differences in the pattern of alcohol consumption between males and females. A total of 24.8% of respondents reported use of marijuana during one’s lifetime, and about 2.5% reported use of marijuana more than 100 times. There were no differences in the pattern of marijuana use between genders. A total of 11.6% of respondents reported use of marijuana during the last 30 days, with patterns of use not differing by gender. Other types of drug use, such as cocaine, sniffed glue, methamphetamines (speed), ecstasy, pills (without doctor’s prescription) was reported by 18.2% of respondents. No differences between genders were found.

**Table 4:** Frequency of health risk behaviors by gender: Alcohol and drug use.

Pattern of alcochol and drug use	Male N(%)	Female N(%)	Total N(%)	Significance (χ^2^, df, p-value*)
**Alcohol use during the last 30 days**				4.301; 3; 0.231
**0**	109 (65.7%)	163 (70.0%)	272 (68.2%)	
**1–9.**	40 (24.1%)	56 (24.0%)	96 (24.1%)	
**10.-19**	12 (6.6%)	12 (5.2%)	23 (5.8%)	
**20 or more**	6 (3.6%)	2 (0.9%)	8 (2.0%)	
**5 or more drinks of alcochol during the last 30 days **				4.512; 3; 0.211
**0**	132 (80.0%)	202 (87.1%)	334 (84.1%)	
**1–9.**	27 (16.4%)	23 (9.9%)	50 (12.6%)	
**10.-19**	3 (1.8%)	5 (2.2%)	8 (2.0%)	
**20 or more**	3 (1.8%)	2 (.9%)	5 (1.3%)	
**Marijuna use during the life **				3.966; 3; 0.265
**0**	127 (76.5%)	173 (74.2%)	300 (75.2%)	
**1–9.**	23 (13.9%)	43 (18.5%)	66 (16.5%)	
**10.-99**	13 (7.8%)	10 (4.3%)	23 (5.8%)	
**100 or more**	3 (1.8%)	7 (3.0%)	10 (2.5%)	
**Marijuna use during the last 30 days **				2.349; 3; 0.503
**0**	146 (88.5%)	204 (88.3%)	350 (88.4%)	
**1–9.**	15 (9.1%)	18 (7.8%)	33 (8.3%)	
**10.-99**	4 (2.4%)	6 (2.6%)	10 (2.5%)	
**20 or more**	0 (.0%)	3 (1.3%)	3 (.8%)	
**Other types of drug use **				0.806; 1; 0.369
**Yes**	26 (15.9%)	46 (19.9%)	72 (18.2%)	
**No**	138 (84.1%)	185 (80.1%)	323 (81.8%)	

*p < .05

### Factors associated with adverse experiences

Overall, ACEs were associated with increased odds of early sex initiation, alcohol use, drug use, and dating violence, although few of these associations were statistically significant. Emotional neglect was the exception, with significantly increased odds observed for all four of the health risk behaviors (Table 5). Emotional abuse was associated with an increased odds of drug abuse (OR = 2.78; 95% CI = 1.31–5.90) and with dating violence (OR = 2.31; 95% CI = 1.10–4.89).

**Table 5:** Interrelatedness of ACEs and health-risk behaviors.

Type of ACE	Early sex ≤16 years		Alcohol use		Drug abuse		Dating violence	
	N	%	N	%	N	%	N	%
Percentage	38	9.5%	127	31.8%	124	31.3%	142	36.3%
Adjusted relative odds*	OR	95%CI	OR	95%CI	OR	95%CI	OR	95%CI
Emotional abuse	1.14	0.36–3.64	0.84	0.38–1.85	2.78	1.31–5.90	2.31	1.1–4.89
Physical abuse	0.81	0.25–2.60	2.46	0.92–6.56	0.65	0.24–1.75	1.70	0.59–4.92
Sexual abuse	3.17	0.93–10.83	0.98	0.36–2.67	1.30	0.43–3.91	2.20	0.72–6.76
Emotional neglect	3.27	1.15–9.33	2.18	1.03–4.64	3.35	1.59–7.06	2.59	1.26–5.31
Physical neglect	1.15	0.35–3.71	0.76	0.29–1.96	0.78	0.32–1.92	1.11	0.42–2.92
Substance abuse in family	1.05	0.34–3.22	1.00	0.44–2.24	2.07	0.92–4.65	1.31	0.56–3.04
Mother treated violently	1.05	0.27–4.05	1.57	0.61–4.04	0.68	0.26–1.80	1.06	0.41–2.76
Family member in prison	1.15	0.16–8.51	3.61	0.95–13.75	4.28	0.93–19.78	4.22	0.999–17.79
Mental illness	0.90	0.21–3.90	2.22	0.85–5.82	1.75	0.62–4.99	0.72	0.25–2.06
Parental divorce or separation	0.61	0.21–1.77	2.43	1.22–4.81	1.45	0.69–3.01	1.58	0.80–3.10

*Odds ratios were adjusted for sex, age, socioeconomic status, residence status, relationship status, and occupational status.

Sexual abuse was marginally associated with increased early sex initiation (OR = 3.2; 95% CI 0.93–10.8). Of the familial ACEs, reporting a family member in prison had the strongest associations with health risk behaviors and was associated with marginally significant increases for alcohol abuse, drug abuse, and dating violence. Parental divorce was significantly associated with alcohol abuse. Compared with participants who had experienced no types of adverse childhood experiences, those who experienced one or more were significantly more likely to be engaged in risky health behaviors (Table 6).

**Table 6:** Adjusted odds ratios of health risk behaviors by number of ACE (and 95% confidence intervals).

No. of experiences	Early sex ≤16 years		Alcohol use		Drug abuse		Dating violence	
	OR*	95%CI	OR	95%CI	OR	95%CI	OR	95%CI
0 (N = 202)	ref		ref		ref		ref	
1 (N = 51)	1.41	0.33–6.04	1.96	0.93–4.16	6.43	2.87–14.40	3.23	1.56–6.67
2 (N = 49)	4.12	1.30–13.14	2.99	1.33–6.74	5.3	2.35–11.95	5.4	2.52–11.57
3 (N = 33)	0.65	0.10–4.13	8.19	3.27–20.51	3.93	1.48–10.44	13.82	5.25–36.41
≥4 (N = 59)	4.65	1.50–14.36	9.18	3.98–21.18	15.85	6.60–38.06	12.68	5.50–29.22

*Odds ratios were adjusted for sex, age, socio-economic status, resident population, relationship status, and occupational status.

## Discussion

Our study showed that 48.7% of respondents had experienced some form of childhood adversity. Emotional neglect was the most common type of adverse childhood experience (25.6%) and was significantly more prevalent among females. Other research from the region suggests that emotional neglect of children is widespread [27,28]. A survey conducted by the In Foundation to examine the experiences of young people in Bosnia and Herzegovina during the war found that 65% of respondents had experienced some form of violence in childhood. The most common types of abuse reported were emotional (62%), physical (58%), witnessing violence (43%), followed by neglect (27%) and sexual abuse (23.4%) [32]. The prevalence of reported adverse events in our study was lower than this survey but still higher than what has been reported in a number of international studies [4,6,13,15,17]. Bosnia and Herzegovina was experiencing a war or post-war period during the time when our participants were children, and war stress and economic insecurity could contribute to parental emotional neglect. However, the association of emotional neglect with parental stress under wartime conditions has not been widely studied.

In this study, we observed a significant level of sexual abuse. A total of 7.1% of respondents reported some type of sexual abuse. Results from studies from the region report that sexual abuse prevalence varied between 6% and 12.9% [27,28,33]. Child sexual abuse was associated with early initiation of sexual experience, indicating that early childhood experiences strongly influence adolescent sexual health. In our study, average age of first sexual intercourse among young people was 18.7 years; 9.5% of respondents had their first sexual experience before the age of 16.

Our study indicated that more than 15% of respondents had witnessed domestic violence. Other reported forms of household stressors included parents divorced or separated (24.1%), substance abuse by a family member (15.1%), mental illness of a family member (7.5%) and a family member in prison (4.2%). According to research on the prevalence of violence against women in Bosnia and Herzegovina from 2013, more than half of women surveyed (47.2%) experienced at least one form of violence from the age of 15, with psychological and physical violence reported by 41.9% and 24.3%, respectively [34].

In the present study, psychological violence was the most common type of partner violence (33.2%), followed by physical (14.0%) and sexual violence (11.5%). A survey of high school students in Tuzla Canton in Bosnia and Herzegovina showed that most adolescents used psychological forms of violence (7–36%), while a smaller proportion of young people physically (4–5%) and sexually (1%) abused a person with whom they had a relationship [35]. About 1 in 10 high school students (9.8%) in the United States reported the experience of physical violence from a partner with whom they were in relationship in the previous 12 months, and this proportion increased by almost 1 in 5 for female students (17.7%) [36]. Another study among young adults in the US found 16.0% of respondents who reported either perpetration of or victimization by physical dating violence before the age of 21 years [15]. These differences could be due to a variety of factors including how the samples were generated, how indicators were defined and measured, and true variation by country. In addition, young adults in different countries may have different comfort levels in reporting their experiences with violence and/or their use of violent tactics. Higher reported behaviors could be an indicator of the openness of the culture in addressing violence as a public health issue.

The results of this study indicate that during the last month before the study alcohol was used by 31.8% of respondents (15.9% reporting 5 or more drinks in a row) and marijuana was used by 11.6% (24.8% indicating ever used) of participants. No differences between genders were found. A research study among young people across Europe reported a significantly higher percentage of respondents using alcohol and drugs, with strong associations observed between sexual risk behavior and substance use. The association between early initiation of sexual activity and using substances was particularly strong among females [22].

Participants reporting childhood emotional neglect were 2.2 times more likely to engage in alcohol use, 3.4 times to abuse drugs, 2.6 times more likely to be victims of dating violence, and 3.3 times more likely to have had early sexual initiation. Emotional abuse increased the odds of drug abuse 2.8 times and dating violence 2.3 times. Parental separation or divorce increased the odds of alcohol use by 2.4 times. Studies from countries in the region, including Macedonia, Romania, and Albania, are consistent with these findings and previous international studies observed that the experience of childhood abuse is associated with participation in risky lifestyles and poor health outcomes in later life [27,28,33].

These findings indicate that early interventions to reduce adverse childhood experiences could have an impact on improving a range of health outcomes in adolescents. Trauma informed care practices, for example, could reduce the negative influence of trauma while adolescents are seeking care from formal agencies [23,24]. Community-level programs that address social networks, engage community agencies, and set a positive tone against risky behaviors have also been successful in low-resource settings [25]. Programs to prevent adverse childhood experiences directly could also have a significant impact in reducing long-term related health sequellae. For example, parenting programs have been found to be effective in several studies [37].

The results of this study are subject to certain limitations. Firstly, the study was conducted in a non-representative sample and as such the results of the study cannot be generalized to the whole population. These data were collected cross-sectionally, therefore, recall of childhood experiences was reported at the same time as the outcome measures. A potential weakness of studies with retrospective reporting of childhood experiences, as it was reported before, is the possibility of recall bias, if those engaging in risky behavior have a greater recall of ACEs [38,39]. Differential recall is a possibility, depending upon the nature and significance of the events.

## Conclusions

Compared with similar studies from the region, our sample included a wider profile of young people. Most previous studies have included students, who represent a higher socioeconomic class than a general clinic population. This is also one of the largest studies on adverse childhood experience conducted in Bosnia and Herzegovina. The results of this study showed a consistent association between adverse experiences in childhood and the probability of engaging in risky lifestyle habits in young adulthood, potentially resulting in poor health outcomes in the long-term.

## Supporting information

S1 Questionnaire Questionnaire in English. (DOCX)

S2 Questionnaire Questionnaire in Bosnian. (DOC)
