# Supplementary material for: Association of adverse childhood experiences and health risk behaviors among young adults visiting a regional primary healthcare center, Federation of Bosnia and Herzegovina
Source: PLoS One. 2018 Mar 29;13(3):e0194439. doi: 10.1371/journal.pone.0194439 (PMC5875750; doi:10.1371/journal.pone.0194439)
Supplement: S1 Questionnaire — (DOCX) [file pone.0194439.s001.docx]

**Epidemiology of sexual and reproductive health of young people and the association with risk factors in the context of family and peer environments**

**The research aims are to collect data concerning sexual and reproductive health of the young people in the Federation of Bosnia and Herzegovina. You are, like other subjects, selected to participate in the study randomly. Your anonymity is guaranteed, and your answers will be available exclusively undersigned researchers for scientific analysis. It is extremely important to complete the questionnaire entirely - honestly answering questions. The questionnaire does not sign, nor record data over which you can later be identified. On average, filling out the questionnaire takes 20 minutes.**

**Thank you for your participation.**

**Mr. sc. dr. Sanjin Musa**

**Dr. Nina Jovanović**

**No#:** !__!__!__!__!

DEPARTMENT: ____________________

| MODUL A GENERAL INFORMATION | | | | | | | | |
| --- | --- | --- | --- | --- | --- | --- | --- | --- |
| Br. | Question | | Response | | | | K | Jump |
| **A1** | Choose sex | | Male  Female | | | 🞎  🞎 | 1  2 |  |
| **A2** | How old are you? | | __________________years | |  | |  |  |
| **A4** | In what kind of place you spend most of your life? | | Less than 10.000 inhabitants  10.001 - 50.000 inhabitants  50.001 - 100.000 inhabitants  100.001 - 500.000 inhabitants  More than 500.000 inhabitants | | 🞎  🞎  🞎  🞎  🞎 | | 1  2  3  4  5 |  |
| **A5** | Assess your financial situation (ie the financial situation of your family): | | Much worse than most other  Slightly worse than most other            Neither better nor worse than most other                   Slightly better than most other                 Much better than most other | | 🞎  🞎  🞎  🞎  🞎 | | 1  2  3  4  5 |  |
| **A6** | Are you currently:  (only one answer) | | Married  ln relationship  Alone  Something else  (please specify): __________________ | | 🞎  🞎  🞎  🞎 | | 1  2  3  4 |  |
| **A7** | Are you raised religiously? | | No     Yes, but not strictly (more formal)  Yes, I'm being asked to respect and implement the principles of religious | | 🞎  🞎  🞎 | | 1  2  3 |  |
| **A8** | Are you currently:  (only one answer) | | Pupil                                                     Student             Employees                                                    Unemployed | 🞎  🞎  🞎  🞎 | | | 1  2  3  4 |  |
| **A9** | What is the highest school level that your parents have completed ? | \|  \| Father \| Mother \| \| --- \| --- \| --- \| \| Incomplete primary school \| 🞎 1 \| 🞎 1 \| \| Primary school \| 🞎 2 \| 🞎 2 \| \| High school \| 🞎 3 \| 🞎 3 \| \| College \| 🞎 4 \| 🞎 4 \| | | | | |  |  |

| **MODULE B** | | | | | | | |
| --- | --- | --- | --- | --- | --- | --- | --- |
| **Following questions are related to behavior linked to sexual and reproductive health** | | | | | | | |
| **No** | Question | **Response** |  | | **K** | | Jump |
| **B1** | Have you ever had sexual intercourse? | Yes  No | 🞎  🞎 | | | 1  2 | B18 |
| **B2** | How old were you when you first sexual intercourse? | __________________________(years) |  | | |  |  |
| **B3** | Did you and your partner use a protection at the first sexual intercourse?  *If you have used more of them, mark all that you used.* | No  Coitus interruptus  Condom  Contraceptive pills.  Natural methods (counting fertile days, measuring basal temperature etc.)  Something else  Be precise ________________________  Don't know | 🞎  🞎  🞎  🞎  🞎  🞎  🞎 | | | 1  2  3  4  5  8  9 |  |
| **B4** | Have you ever had sex with someone you just met, with someone of whom you knew very little or nothing? | Yes  No | 🞎  🞎 | | | 1  2 |  |
| **B5** | With how many people have had sexual intercourse in the past 12 months?  *(If you have not had sexual intercourse, write 0)* | (write number)____________________ | | | |  |  |
| **B6** | From your first sexual intercourse until today, with how many people you had sexual intercourse? | (write number)____________________ | | | |  |  |
| **B7** | Did you and your partner use a protection during the last sexual intercourse ?  *If you have used more of them, mark all that you used.* | No  Coitus interruptus  Condom  Contraceptive pills.  Natural methods (counting fertile days, measuring basal temperature etc.)  Something else  Be precise ________________________  Don't know | | 🞎  🞎  🞎  🞎  🞎  🞎  🞎 | | 1  2  3  4  5  8  9 |  |
| **B8** | If you did not use a condom during last intercourse, what was the main reason ?  *Only one answer.* | I do not want to use condoms  Partner does not want to use a condom  Something else  Be precize___________________ | | 🞎  🞎  🞎 | | 1  2  3 |  |
| **B9** | If you remember the sexual intercourse that you had during the past year, how often did you use a condom? | In that period I did not have sex  never  rarely  sometimes  often  Every time (always) | | 🞎  🞎  🞎  🞎  🞎  🞎 | | 1  2  3  4  5  6 |  |
| **B10** | What do you think, what is the risk that you personally get infected with HIV / AIDS?  *Circle the appropriate number: number 1 indicates minimal risk, and the number 10 means extremely high risk of infection:* | 1 2 3 4 5 6 7 8 9 10 | | | |  |  |
| **B11** | What do you think, what is the risk that you personally get infected with other STI?  *Circle the appropriate number: number 1 indicates minimal risk, and the number 10 means extremely high risk of infection:* | 1 2 3 4 5 6 7 8 9 10 | | | |  |  |
| **B12** | Have you ever been diagnosed with a sexually transmitted disease? (by a doctor) | Yes 🞎  No 🞎 | | | | 1  2 |  |
| **B13** | Which a sexually transmitted disease ? | Genital herpes 🞎  Trichomonas 🞎  Gonorrhea 🞎  Syphilis 🞎  Chlamydia 🞎  Human papillomavirus 🞎  Candidiasis 🞎  Other___________________ | | | | 1  2  3  4  5  6  7 |  |
| **B14** | Have you ever tested for HIV? | Yes 🞎  No 🞎 | | | | 1  2 |  |
| The following questions are to be completed by female respondents only | | | | | | | |
| HPV vaccine | | | | | | | |
| **B15** | Have you ever heard for HPV vaccine (human papilloma virus) | Yes, I would like to get vaccinated 🞎  Yes, but I would’t like to get vaccinated 🞎  Never heard 🞎 | | | | 1  2  3 |  |

| MODUL C | | | | |
| --- | --- | --- | --- | --- |
| The next three questions relate to your experience with dating violence | | | | |
| **No** | Question | **Response** | **K** | Jump |
| **C1** | Have you ever been in a relationship with someone who hit you, slap you or physically hurt or threatened to hit or slap you, destroyed something that belongs to you? | Yes 🞎  No 🞎 | 1  2 |  |
| **C2** | Have you ever been in a relationship with someone who forced you to have sexual intercourse arguing with you or threating you with physical force? | Yes 🞎  No 🞎 | 1  2 |  |
| **C3** | Have you ever been in a relationship with someone who try to had control a your behaviour by always checking, you, called you names,said things to hurt you, shouted at you, made unwanted calls, text messages or emails? | Yes 🞎  No 🞎 | 1  2 |  |

| MODUL D | | | | |
| --- | --- | --- | --- | --- |
| The following section refers to the use of alcohol and drugs | | | | |
| No | Question | Response | K | Jump |
| **D1** | During the past 30 days, on how many days did you have at least one drink of alcohol? | 0 days 🞎  1 to 9 days 🞎  10 to 19 days 🞎  20 or more days 🞎 | 1  2  3  4 |  |
| **D2** | During the past 30 days, on how many days did you have 5 or more drinks of alcohol in a row, that is, within a couple of hours? | 0 days 🞎  1 to 9 days 🞎  10 to 19 days 🞎  20 or more days 🞎 | 1  2  3  4 |  |
| **D3** | During your life, how many times did you use marijuana? | 0 times 🞎  1 to 9 times 🞎  10 to 99 times 🞎  More then 100 times 🞎 | 1  2  3  4 |  |
| **D4** | During the last month, how many times did you use marijuana? | 0 times 🞎  1 to 9 times 🞎  10 to 19 times 🞎  20 or more times 🞎 | 1  2  3  4 |  |
| **D5** | Have you ever used drugs intravenously? | Yes 🞎  No 🞎 | 1  2 |  |
| **D6** | Have you ever used other types of drugs: cocaine, sniffed glue, methamphetamines (speed), ecstasy, pills (without doctor’s prescription) ? | Yes 🞎  No 🞎 | 1  2 |  |

| MODUL E Thinking about your experiences while growing up, please check if you had any of these experiences. | | | | | |
| --- | --- | --- | --- | --- | --- |
| **No** | Question | **Response** | **K** | | Jump |
| **E1** | How often parent or other adult in your household swear at you, insult you, put your down or humiliate you? | Never rarely sometimes often very often  🞎 0 🞎 1 🞎 2 🞎 3 🞎 4 | |  |  |
| **E2** | How often did a parent or other adult in your household act in a way that made you afraid that you might be physically hurt? | Never rarely sometimes often very often  🞎 0 🞎 1 🞎 2 🞎 3 🞎 4 | |  |  |
| **E3** | How often did a parent or other adult in the household push, grab, slap, or throw something at you? | Never rarely sometimes often very often  🞎 0 🞎 1 🞎 2 🞎 3 🞎 4 | |  |  |
| **E4** | Did an adult or person at least 5 years older than you ever touch or fondle you or have you touch their body in a sexual way? | Yes 🞎  No 🞎 | | 1  2 |  |
| **E5** | Did an adult or person at least 5 years older than you attempt or actually have oral, anal, or vaginal intercourse with you? | Yes 🞎  No 🞎 | | 1  2 |  |
| **E6** | How often did you feel that no one in your family loved you or thought you were important or special? | Never rarely sometimes often very often  🞎 0 🞎 1 🞎 2 🞎 3 🞎 4 | |  |  |
| **E7** | How often did you feel that you didn’t have enough to eat, had to wear dirty clothes, or had no one to protect you? | Never rarely sometimes often very often  🞎 0 🞎 1 🞎 2 🞎 3 🞎 4 | |  |  |
| **E8** | How often did you feel that your family didn’t look out for each other, feel close to each other, or support each other? | Never rarely sometimes often very often  🞎 0 🞎 1 🞎 2 🞎 3 🞎 4 | |  |  |
| **E9** | Were your parents ever separated or divorced? | Yes 🞎  No 🞎 | | 1  2 |  |
| **E10** | Was your mother or stepmother often or very often pushed, grabbed, slapped, or had something thrown at her? | Never rarely sometimes often very often  🞎 0 🞎 1 🞎 2 🞎 3 🞎 4 | |  |  |
| **E11** | Was your mother or stepmother ever repeatedly hit at least a few minutes or threatened with a gun or knife? | Never rarely sometimes often very often  🞎 0 🞎 1 🞎 2 🞎 3 🞎 4 | |  |  |
| **E12** | Did you ever live with someone who was a problem drinker or alcoholic or who used street drugs? | Yes 🞎  No 🞎 | | 1  2 |  |
| **E13** | Was a household member depressed or mentally ill, or did a household member attempt or commit suicide? | Yes 🞎  No 🞎 | | 1  2 |  |
| **E14** | Did a household member go to prison? | Yes 🞎  No 🞎 | | 1  2 |  |

**Thank you for participating in the survey!**
