# Supplementary material for: Association of adverse childhood experiences and health risk behaviors among young adults visiting a regional primary healthcare center, Federation of Bosnia and Herzegovina
Source: PLoS One. 2018 Mar 29;13(3):e0194439. doi: 10.1371/journal.pone.0194439 (PMC5875750; doi:10.1371/journal.pone.0194439)
Supplement: S2 Questionnaire — (DOC) [file pone.0194439.s002.doc]

| **MODUL A: OSNOVNI PODACI** | | | | | | | | |
| --- | --- | --- | --- | --- | --- | --- | --- | --- |
| **Br.** | **Pitanje** | | **Odgovor** | | | | **K** | **Preskok** |
| **A1** | OZNAČITI SPOL | | Muški  Ženski | | | 🞎  🞎 | 1  2 |  |
| **A2** | Koliko imate godina? | | __________________godina | |  | |  |  |
| **A4** | U kakvom ste mjestu proveli najveći dio svog života?  *(grad Zenica ima od 100.000-500.000 stanovnika)* | | Manje od 10.000 stanovnika  Od 10.001 do 50.000 stanovnika  Od 50.001 do 100.000 stanovnika  Od 100.001 do 500.000 stanovnika  Više od 500.000 stanovnika | | 🞎  🞎  🞎  🞎  🞎 | | 1  2  3  4  5 |  |
| **A5** | Procijenite Vaše imovinsko stanje (odnosno imovinsko stanje Vaše obitelji): | | Mnogo gore od većine drugih  Nešto gore od većine drugih  Ni bolje, ni gore od većine drugih  Nešto bolje od većine drugih  Mnogo bolje od većine drugih | | 🞎  🞎  🞎  🞎  🞎 | | 1  2  3  4  5 |  |
| **A6** | Jeste li trenutno u:  *(samo jedan odgovor)* | | Braku  Vezi  Sama/sam  Nešto drugo  (navedite što):__________________ | | 🞎  🞎  🞎  🞎 | | 1  2  3  4 |  |
| **A7** | Jesu li Vas u obitelji odgajali u vjerskom duhu? | | Ne  Da, ali ne striktno (više formalno)  Da, od mene se tražilo da poštujem i provodim vjerska načela | | 🞎  🞎  🞎 | | 1  2  3 |  |
| **A8** | Jeste li trenutno:  *(jedan odgovor)* | | Učenik/ca  Student/ica  Zaposleni (uključujući i „rad na crno“)  Nezaposleni | 🞎  🞎  🞎  🞎 | | | 1  2  3  4 |  |
| **A9** | Koja je najviša škola koju su završili Vaši roditelji ? | |  | Otac | Majka | | --- | --- | --- | | Nepotpuna osnovna škola | 🞎 1 | 🞎 1 | | Osnovna škola | 🞎 2 | 🞎 2 | | Srednja škola | 🞎 3 | 🞎 3 | | Viša škola ili fakultet | 🞎 4 | 🞎 4 | | | | | |  |  |

| **MODUL B** | | | | | | | |
| --- | --- | --- | --- | --- | --- | --- | --- |
| ***Sljedeća pitanja tiču se ponašanja vezanog za seksualno i reproduktivno zdravlje*** | | | | | | | |
|  | **Pitanje** | **Odgovor** |  | | **K** | | **Preskok** |
| **B1** | Jeste li ikada imali seksualni odnos? | Da  Ne | 🞎  🞎 | | | 1  2 | B18 |
| **B2** | Koliko ste imali godina pri prvom seksualnom odnosu ? | __________________________(godina) |  | | |  |  |
| **B3** | Jeste li pri prvom seksualnom odnosu Vi i Vaš partner/partnerica koristili neko sredstvo zaštite ?  *Ako ste koristili više njih, zaokružite sva sredstva koja ste koristili****.*** | Ne  Prekinuti snošaj  Kondom (prezervativ)  Kontracepcijske pilule.  Prirodne metode (brojanje plodnih dana, mjerenje bazalne temperature itd.)  Nešto drugo  Navesti ________________________  Ne znam | 🞎  🞎  🞎  🞎  🞎  🞎  🞎 | | | 1  2  3  4  5  8  9 |  |
| **B4** | Jeste li ikada imali seksualni odnos s osobom koju ste tek upoznali, to jest s nekim o kome ste znali vrlo malo ili ništa ? | Da  Ne | 🞎  🞎 | | | 1  2 |  |
| **B5** | S koliko ste osoba imali seksualni odnos u posljednjih 12 mjeseci ?  *(Ako niste imali seksualni odnos, upišite 0)* | (upišite broj)____________________ | | | |  |  |
| **B6** | Od Vašeg prvog seksualnog odnosa do danas, s koliko ste osoba ukupno imali seksualni odnos ? | (upišite broj)____________________ | | | |  |  |
| **B7** | Jeste li koristili neko sredstvo zaštite pri posljednjem seksualnom odnosu ?  *(Ako ste koristili više njih, zaokružite sva sredstva koja ste koristili.)* | Ne  Prekinuti snošaj  Kondom (prezervativ)  Kontracepcijske pilule  Prirodne metode (brojanje plodnih dana, mjerenje bazalne temperature itd.)  Nešto drugo  Navesti ____________________  Ne znam | | 🞎  🞎  🞎  🞎  🞎  🞎  🞎 | | 1  2  3  4  5  88  89 |  |
| **B8** | Ako pri posljednjem odnosu niste koristili kondom (prezervativ), koji je bio glavni razlog?  *Samo jedan odgovor.* | Ne želim koristiti kondome  Partner/partnerica ne želi koristiti kondom  Nešto drugo  Navesti_________________ | | 🞎  🞎  🞎 | | 1  2  3 |  |
| **B9** | Ako se prisjetite seksualnih odnosa koje ste imali tijekom posljednjih *godinu* dana, koliko ste često koristili kondom ? | U tom periodu nisam imao/la seks.odnose  Nikada  Rijetko  Ponekad  Često  Svaki put (uvijek) | | 🞎  🞎  🞎  🞎  🞎  🞎 | | 1  2  3  4  5  6 |  |
| **B10** | Što mislite, kolika je mogućnost (tj. rizik) da se Vi osobno zarazite HIV/ AIDS-om?  *Zaokružite odgovarajući broj: brojka 1 označava zanemariv rizik, a brojka 10 izrazito visok rizik zaraze:* | 1 2 3 4 5 6 7 8 9 10 | | | |  |  |
| **B11** | Što mislite, kolika je mogućnost (tj. rizik) da se Vi osobno zarazite nekom drugom spolno prenosivom bolesti?  *Zaokružite odgovarajući broj: brojka 1 označava zanemariv rizik, a brojka 10 izrazito visok rizik zaraze:* | 1 2 3 4 5 6 7 8 9 10 | | | |  |  |
| **B12** | Je li Vam ikada dijagnosticirana neka spolno prenosiva bolest ? (od strane ljekara) | Da 🞎  Ne 🞎 | | | | 1  2 |  |
| **B13** | Koja spolno prenosiva bolest ? | Genitalni herpes 🞎  Trihomonas 🞎  Gonoreja 🞎  Sifilis 🞎  Klamidija 🞎  Humani papiloma virus 🞎  Kandidijaza 🞎  Drugo___________________ | | | | 1  2  3  4  5  6  7 |  |
| **B14** | Jeste li se ikada testirali na HIV? | Da 🞎  Ne 🞎 | | | | 1  2 |  |
| **Na sljedeća pitanje odgovaraju samo djevojke** | | | | | | | |
| **HPV vakcina** | | | | | | | |
| **B15** | Jeste li čuli za HPV vakcinu (vakcinu protiv humanog papilloma virusa)? | Da, željela bih se vakcinisati 🞎  Da, ali se ne bih vakcinisala 🞎  Nisam čula 🞎 | | | | 1  2  3 |  |

| **MODUL C** | | | | |
| --- | --- | --- | --- | --- |
| **Sljedeća tri pitanja odnose se na Vaše iskustvo sa nasiljem u vezi** | | | | |
| **Br** | **Pitanje** | **Odgovor** | **K** | **Preskok** |
| **C1** | Jeste li ikada bili u vezi sa osobom koja Vas je: udarila, ošamarila ili fizički ozljedila ili prijetila, uništila nešto što Vama pripada ? | Da 🞎  Ne 🞎 | 1  2 |  |
| **C2** | Jeste li ikada bili u vezi sa osobom koja Vas je: prisilila na seksualni odnos raspravom ili prijetnjom fizičkom silom ? | Da 🞎  Ne 🞎 | 1  2 |  |
| **C3** | Jeste li ikada bili u vezi sa osobom koja Vas je: pokušala kontolirati stalnim provjeravanjem, nazivala Vas različitim imenima, govorila stvari da bi Vas povrijedila, derala se na Vas, telefonski uznemiravala, slala poruke ili e-mailove ? | Da 🞎  Ne 🞎 | 1  2 |  |

| **MODUL D** | | | | |
| --- | --- | --- | --- | --- |
| **Sljedeća sekcija se odnosi na korištenje alkohola i droga** | | | | |
| **Br.** | **Pitanje** | **Odgovor** | **K** | **Preskok** |
| **D1** | Tokom posljednjeg mjeseca, koliko je bilo dana kada ste popili barem jedno alkoholno piće ? | 0 dana 🞎  1 do 9 dana 🞎  10 do 19 dana 🞎  20 ili više dana 🞎 | 1  2  3  4 |  |
| **D2** | Tokom posljednjeg mjeseca, koliko je bilo dana kada ste popili više od 5 alkoholnih pića u nekoliko sati ? | 0 dana 🞎  1 do 9 dana 🞎  10 do 19 dana 🞎  20 ili više dana 🞎 | 1  2  3  4 |  |
| **D3** | Tokom života, koliko ste puta koristili marihuanu? | 0 puta 🞎  1 do 9 puta 🞎  10 do 99 puta 🞎  Više od 100 puta 🞎 | 1  2  3  4 |  |
| **D4** | Tokom posljednjeg mjeseca, koliko ste puta koristili marihuanu? | 0 puta 🞎  1 do 9 puta 🞎  9 do 19 puta 🞎  20 ili više puta 🞎 | 1  2  3  4 |  |
| **D5** | Jeste li ikada intravenozno koristili drogu ? | Da 🞎  Ne 🞎 | 1  2 |  |
| **D6** | Jeste li ikada koristili druge vrste droge: kokain, ljepilo, metamfetamine (speed), ekstazi, tabelete (koje nije prepisao ljekar) ? | Da 🞎  Ne 🞎 | 1  2 |  |

| **MODUL E** **Prisjećajući se iskustava tokom odrastanja, molimo Vas prisjetite se da li ste imali neko od navedenih iskustava.** | | | | | |
| --- | --- | --- | --- | --- | --- |
| **Br** | **Pitanje** | **Odgovor** | **K** | | **Preskok** |
| **E1** | Koliko često Vas je roditelj ili neka druga odrasla osoba iz domaćinstva psovala, vrijeđala ili ponižavala? | Nikad rijetko ponekad često jako često  🞎 0 🞎 1 🞎 2 🞎 3 🞎 4 | |  |  |
| **E2** | Koliko često se roditelj ili neka druga odrasla osoba iz domaćinstva ponašala na način da ste bili zabrinuti da biste mogli biti ozljeđeni? | Nikad rijetko ponekad često jako često  🞎 0 🞎 1 🞎 2 🞎 3 🞎 4 | |  |  |
| **E3** | Koliko često Vas je roditelj ili neka druga odrasla osoba iz domaćinstva gurnula, ogrebala, udarila ili bacila nešto na vas ? | Nikad rijetko ponekad često jako često  🞎 0 🞎 1 🞎 2 🞎 3 🞎 4 | |  |  |
| **E4** | Da li Vas je odrasla osoba ili osoba starija od Vas najmanje 5 godina, ikada dodirnula ili pomilovala ili ste Vi dodirnuli njeno tijelo u seksualnom smislu? | Da 🞎  Ne 🞎 | | 1  2 |  |
| **E5** | Da li je odrasla osoba ili osoba starija od Vas najmanje 5 godina pokušala ili imala oralni, analni ili vaginalni odnos sa Vama? | Da 🞎  Ne 🞎 | | 1  2 |  |
| **E6** | Koliko često se osjećali da Vas niko u porodici ne voli ili ne misli da ste važni ili posebni? | Nikad rijetko ponekad često jako često  🞎 0 🞎 1 🞎 2 🞎 3 🞎 4 | |  |  |
| **E7** | Koliko često ste osjećali da nemate dovoljno za jesti, da morate nositi prljavu odjeću ili da nema nikoga da Vas zaštiti? | Nikad rijetko ponekad često jako često  🞎 0 🞎 1 🞎 2 🞎 3 🞎 4 | |  |  |
| **E8** | Koliko često ste osjećali da se članovi Vaše obitelji ne brinu jedni za druge, ne osjećaju bliskim ili ne pružaju podršku jedni drugima. | Nikad rijetko ponekad često jako često  🞎 0 🞎 1 🞎 2 🞎 3 🞎 4 | |  |  |
| **E9** | Jesu li ikada Vaši roditelji živjeli razdvojeno ili su se razveli ? | Da 🞎  Ne 🞎 | | 1  2 |  |
| **E10** | Koliko je često Vaša majka ili maćeha bila odgurivana, ogrebana, udarana ili pogođena nečim čvrstim ? | Nikad rijetko ponekad često jako često  🞎 0 🞎 1 🞎 2 🞎 3 🞎 4 | |  |  |
| **E11** | Koliko je često Vaša majka ili maćeha bila ponavljano udarana najmanje 5 minuta ili joj je bilo prijećeno nožem ili pištoljem ? | Nikad rijetko ponekad često jako često  🞎 0 🞎 1 🞎 2 🞎 3 🞎 4 | |  |  |
| **E12** | Jeste li ikada živjeli sa nekim ko je imao problema sa alkoholizmom ili korištenjem droge? | Da 🞎  Ne 🞎 | | 1  2 |  |
| **E13** | Je li neki član Vašeg domaćinstva bolovao od depresije ili nekog drugog mentalnog oboljenja, ili je pokušao ili počinio samoubistvo ? | Da 🞎  Ne 🞎 | | 1  2 |  |
| **E14** | Je li neki član Vašeg domaćinstva bio u zatvoru ? | Da 🞎  Ne 🞎 | | 1  2 |  |

**Hvala na sudjelovanju u istraživanju!**
